# Blood Pressure Target in Acute Stroke to Reduce HemorrhaGe After Endovascular Therapy: The Randomized BP TARGET Study Protocol

**DOI:** 10.3389/fneur.2020.00480

**Published:** 2020-06-19

**Authors:** Mikael Mazighi, Julien Labreuche, Sebastien Richard, Benjamin Gory, Bertrand Lapergue, Igor Sibon, Jerome Berge, Jean-Marc Olivot, Peggy Reiner, Emmanuel Houdart, Joseph Broderick, Alain Duhammel, Benjamin Maier, Amélie Yavchitz, Laurence Salomon, Michael Obadia, Raphael Blanc, Julien Savatovsky, Michel Piotin

**Affiliations:** ^1^Department of Interventional Neuroradiology, Fondation Ophtalmologique Adolphe de Rothschild Hospital, Paris, France; ^2^Laboratory of Vascular Translational Science, U1148 Institut National de la Santé et de la Recherche Médicale (INSERM), Paris, France; ^3^Paris University, Paris, France; ^4^DHU NeuroVasc, Paris, France; ^5^University of Lille, CHU Lille, EA 2694, Santé Publique: Épidémiologie et Qualité des Soins, Lille, France; ^6^Department of Neurology, Stroke Unit, University Hospital of Nancy, Nancy, France; ^7^INSERM U1116, Nancy, France; ^8^Department of Diagnostic and Therapeutic Neuroradiology, University Hospital of Nancy, Nancy, France; ^9^INSERM U1254, Nancy, France; ^10^Division of Neurology, Department of Neurology, Stroke Center, Foch Hospital, University Versailles Saint-Quentin en Yvelines, Suresnes, France; ^11^Pôle des Neurosciences Cliniques, CHU Bordeaux, Bordeaux, France; ^12^Interventional and Diagnostic Neuroradiology Department, Bordeaux University Hospital, Bordeaux, France; ^13^Stroke Unit, Toulouse University Hospital, Toulouse, France; ^14^Department of Neurology, Lariboisière Hospital, Paris, France; ^15^Department of Neuroradiology, Lariboisière Hospital, Paris, France; ^16^Department of Neurology and Rehabilitation Medicine, University of Cincinnati College of Medicine, Cincinnati, OH, United States; ^17^Department of Clinical Research, Fondation Ophtalmologique Adolphe de Rothschild Hospital, Paris, France; ^18^Stroke Unit, Fondation Ophtalmologique Adolphe de Rothschild Hospital, Paris, France; ^19^Department of Neuroradiology, Fondation Ophtalmologique Adolphe de Rothschild Hospital, Paris, France

**Keywords:** acute ischemic stroke, blood pressure, intracranial hemorrhage, mechanical thrombectomy, randomized controlled trial

## Abstract

**Background:** High systolic blood pressure (BP) is associated with an increased risk of intracranial hemorrhage (ICH) in patients undergoing reperfusion therapy. However, there are no data from randomized trials to guide BP management after reperfusion following endovascular therapy (EVT) for patients with acute ischemic stroke (AIS) with large vessel occlusion (LVO). The objective is to evaluate if BP control with a target of 100–129 mmHg systolic BP (“tight” SBP control) can reduce ICH as compared to 130–185 mmHg (“usual” SBP control) in AIS participants after reperfusion by EVT.

**Methods:** The BP TARGET trial is a multicenter, prospective, randomized, controlled, open-label, blinded endpoint clinical trial. AIS participants with LVO experiencing successful reperfusion are randomly assigned, in a 1:1 ratio, to have a “tight” SBP control (100–129 mmHg) or a conservative SBP control (130–185 mmHg) during the following 24–36 h. The primary outcome is the rate of ICH (either symptomatic or asymptomatic) on follow-up CT scan at 24–36 h. Secondary outcomes include the rate of the symptomatic ICH, the overall distribution of the modified Rankin Scale (mRS) at 90 days, favorable outcome (90–day mRs 0–2), infarct volume at follow-up CT scan at 24–36 h, change in National Institute of Health Stroke Scale at 24 h, and all-cause mortality at 90 days.

**Conclusion:** This is the first randomized trial directly comparing the efficacy of different SBP targets after EVT reperfusion. This prospective trial aims to determine whether a “tight” SBP control after EVT reperfusion can reduce the risk of ICH.

## Introduction and Rationale

High blood pressure (BP) in the setting of acute ischemic stroke (AIS), defined by a systolic BP (SBP) >140 mmHg and diastolic BP (DBP) >90 mmHg (1, 2), occurs in up to 50% of patients and is a predictor of unfavorable outcome (3–5). High BP is associated with an increased risk of intracranial hemorrhage (ICH) in AIS patients treated with alteplase (6). In patients eligible for reperfusion therapies, such as alteplase, BP should be below 185/110 mmHg prior to initiation of therapy (7). In the Safe Implementation of Thrombolysis in Stroke-MOnitoring STudy (SITS-MOST) registry, SBP had a linear relationship with the ICH risk (i.e., the risk increases for high SBP values) (6). High BP values also seem to contribute to the prognosis of AIS patients with large vessel occlusions (LVO) of the anterior circulation treated with endovascular therapy (EVT). Available evidence, in EVT-treated patients, shows that mortality increases for lower and higher baseline SBP values following a U-shaped relationship (8), with a nadir at 157 mmHg (9). In addition, higher SBP peak values independently correlate with unfavorable outcome and a higher ICH rate within 48 h after EVT (8–13). Despite high reperfusion rates and a strong benefit of EVT, more than 50% of patients will remain disabled (14). The lack of clinical recovery may be a consequence, at least in part, of reperfusion injuries including ICH.

Current international guidelines do not discriminate BP management between patients treated with IVT alone and patients treated with EVT and IVT and propose the same BP threshold. Recently, a substantial number of observational studies have begun to shed light on the association between higher post-EVT BP values (SBP >140 or 160 mmHg) and ICH or worse functional outcomes according to the recanalization score (i.e., TICI < 2B vs. TICI ≥ 2B), but with conflicting results (12, 13). Given the lack of data from a randomized controlled trial, an online survey regarding post-EVT BP management was performed across institutions in the United States (StrokeNet Sites)(15). In recanalized patients after EVT, most institutions (36%) seemed to target an SBP in the range of 120–139 mmHg and allow permissive hypertension in nonrecanalized patients.

Taken together, these data underline the need of a randomized controlled trial assessing different BP targets after EVT reperfusion. The BP TARGET trial aims to assess the efficacy of a “tight” BP lowering control (with a target 100–129 mmHg for SBP) versus a more conservative BP control (SBP 130–185 mmHg) following 24 h after reperfusion by EVT in AIS patients.

## Methods

### Design

BP TARGET is a prospective, randomized, multicenter, controlled, open-label, with blinded endpoint design (PROBE). This trial, funded by the French government, is conducted in four high-volume comprehensive stroke centers in France.

Enrollment into the BP TARGET study started in June 2017 and is projected to be completed by December 2019. The data collection phase of study is expected to be completed by January 2020. The study, which is registered with ClinicalTrials.gov (ClinicalTrials.gov Identifier: NCT03160677), is conducted in accordance with the Declaration of Helsinki and Good Clinical Practice. Research ethics approval was obtained from the Comité de Protection des Personnes Sud Méditerranée IV on March 14, 2017. Data collection during the study is reported in Table 1 and carried out using an electronic case report form (eCRF), developed using Cleanfile® software.

**Table 1:** Data collection during the study.

	**Inclusion**	**24-h follow-up**	**72-h follow-up**	**3-month follow-up**
Eligibility criteria	✓			
Informed Consent	✓			
Randomization	✓			
Clinical examination	✓			
mRS score	✓			✓^1^
NIHSS score	✓	✓		
Concomitant Medications	✓	✓		✓
CT scan		✓	✓^2^	
Adverse Event assessment		✓		✓

1 *Must be completed by a BLINDED stroke study team member. CT/CTA or MRI/MRA are required at baseline, and any time there is a neurological deterioration (a change in NIHSS of 4 points or more) or hemorrhage*.

2 *Performed if CT at 24 hours shows a hyperdensity. CT, computed tomography, mRS, modified Rankin Scale; NIHSS, National Institute of Health Stroke Scale*.

### Patient Population

Eligible participants have an AIS due to a large vessel occlusion (LVO) of the anterior circulation [i.e., internal carotid and/or proximal middle cerebral artery (M1) occlusions] and have reperfusion by EVT defined as modified Thrombolysis In Cerebral Infarction (mTICI) of 2b or 3.

### Baseline Eligibility Evaluation

A written consent form must be signed by the participant or legal representative before randomization. Emergency consent procedure may be considered if consent by the participant or a proxy is not possible. In this situation, the participant's consent confirmation is still required and gathered when the participant is able to consent by him- or herself. Reperfusion will be evaluated at the end of the procedure and defined by an mTICI of 2b or 3. For eligibility, SBP needs to be over 130 mmHg during the hour following the intervention. Detailed inclusion and exclusion criteria are in Table 2.

**Table 2:** Inclusion and exclusion criteria.

**Inclusion**	**Exclusion**
• Age 18 and older • Clinical diagnosis of AIS in the anterior circulation • Neuroimaging demonstrates large vessel proximal occlusion (ICA and/or MCA -M1) • With or without IV thrombolysis • Consenting requirements met according to French laws	• Acute ischemic stroke involving posterior circulation (vertebrobasilar occlusion) • Allergy to radiographic contrast agents. • Disability prior to the stroke (mRS >3) • Pregnant or breastfeeding women • Severe or fatal comorbidities that will likely prevent improvement or follow-up or that will render the procedure unlikely to benefit the patient • Under legal protection • No affiliation to a social security scheme • Opposition of the patient or their family

*ICA, internal carotid artery; IV, intravenous; MCA, middle cerebral artery; mRS, modified Rankin Scale*.

### Randomization

**Figure 1:**
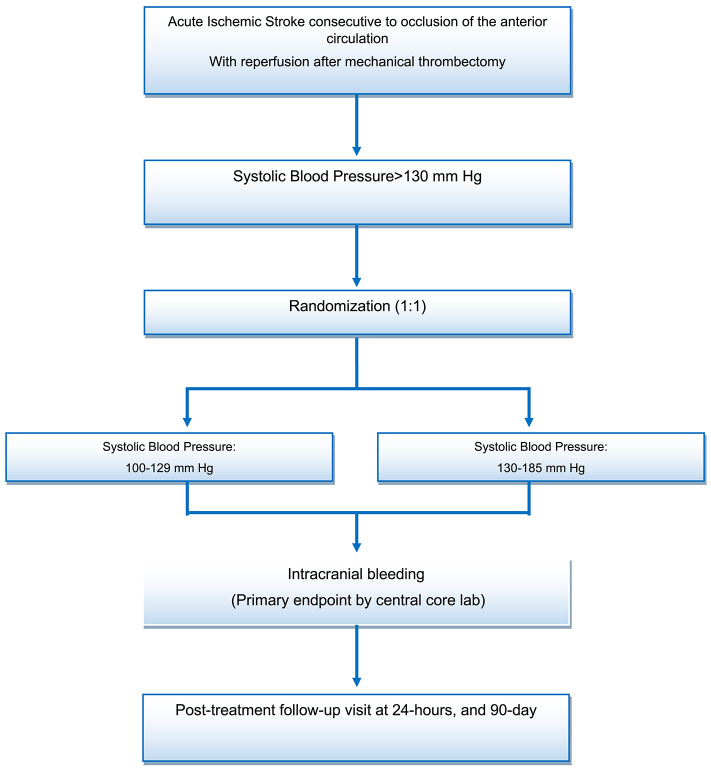
Patient flow diagram.

During the first hour following reperfusion, participants will be randomly allocated in a one-to-one ratio to a “tight” BP control, defined as a target SBP between 100 and 129 mmHg (experimental arm), or a standard BP control, defined as target SBP level between 130 and 185 mmHg (control arm), during the following 24 h (Figure 1). The randomization sequence will be provided by an independent statistician (who did not take part in assessing the participants at any point in the study) using computer-generated random numbers with block sizes of four and stratification by center and IV alteplase therapy use prior to EVT. The block size information is not specified in the protocol to ensure that vascular neurologists and interventional neuroradiologists will be not able to anticipate treatment arm assignment. The randomization sequence is implemented in the eCRF system to ensure a centralized real-time randomization procedure. Participants are enrolled and randomized by vascular neurologists or interventional neuroradiologists.

### Interventions

Once the subject is randomized, the BP target has to be obtained within 1 h.

#### Experimental Arm: Target SBP 100–129 mmHg

Participants allocated to this arm will achieve an SBP target of 100–129 mmHg during the following 24 h. BP measurement is performed with automatic sphygmomanometers and conducted for 24 h, based on the guidelines (16), as follows: every 15 min for 2 h from the start of alteplase therapy, then every 30 min for 6 h, and then every hour for the remaining 16 h. Investigators are free to choose antihypertensive medications to achieve the target BP based on their current practice.

#### Control Arm: Target SBP 130–185 mmHg

Participants allocated to this arm will achieve an SBP target of 130–185 mmHg during the following 24–h, following the same protocol, as described in the preceding text.

### Procedure Care and Follow-up

Standardization of postprocedure medical management in both arms will be conducted according to the European guidelines (17). Neurological and functional exams will be performed [National Institute of health Stroke Scale (NIHSS) and modified Rankin Scale (mRS)] at baseline, 24 (± 12) h, and 3 months (mRS only for the latter timepoint).

Follow-up imaging (i.e., noncontrast CT scan) will be performed at 24 (±12) h after randomization. If a hyperdensity potentially consistent with ICH is documented on CT at 24–36 h, an additional CT is conducted at 72 h to confirm ICH and rule out iodine extravasation. In addition, any neurological deterioration will be evaluated by urgent CT scan and other evaluations as indicated according to the investigator best practice. ICH will be categorized as petechial hemorrhage or hematoma according to the Heidelberg classification (18); a symptomatic ICH will be defined as 24-h CT evidence of bleeding associated with a 4-point or more worsening of the NIHSS according to the European Cooperative Acute Stroke Study (ECASS) III classification (19).

Follow-up examination will be done at 3 months to assess the mRS. This assessment will be performed by certified research nurses, unaware of the group assignments, during face-to-face interviews, or via telephone survey.

For the primary outcome, a central imaging core laboratory, not involved in subjects' management and blinded to treatment allocation, will review the CT imaging. In cases of disagreement between the two assessors (JS, FD), a centralized neuroradiologist (JCS) will review the CT imaging and will decide on the primary endpoint value. Serious adverse events (SAEs) and procedure-related complications (e.g., symptomatic ICH and hypotensive events below 80 mmHg) will be adjudicated by three members of the Data and Safety Monitoring Board (DSMB), blinded to treatment arm.

### Outcomes

#### Primary Outcome

The primary outcome is the rate of subjects with ICH on CT at 24–36 h.

#### Secondary Outcomes

The secondary clinical efficacy outcomes are the disability assessed by overall mRS distribution at 90 days (shift analysis combining scores of 5 and 6), the 3-month favorable functional outcome at 90 days as defined by a mRS 0–2, the 90-day excellent function outcome as defined by a mRS 0–1, the stroke volume as measured by CT at 24–36 h after EVT; stroke volume at 24–36 h, and the change in NIHSS at 24 h.

Safety outcomes are symptomatic ICH defined by an increase of more than 4 points on the NIHSS, parenchymal hematoma type 2, and all-cause mortality at 90 days.

Feasibility outcome is the rate of patients with mean SBP level during BP management <130 mmHg in the experimental group.

### Independent Data Safety Monitoring Board

The purpose of the Independent Data Safety Monitoring Board (DSMB) is to review the unmasked accumulated safety data. The DSMB is composed of two stroke neurologists, one interventional neuroradiologist, and one methodologist, who are not participating in the study and are not affiliated with the sponsor. The role of the DSMB is to review the occurrence of AEs, and it will ensure the balance of complication rates between arms throughout the study by making recommendations to the sponsor.

### Data Collection

The entire study is conducted using electronic case report forms (eCRFs), where all clinical data on enrolled subjects are data entered (single-keyed) by the site personnel. The eCRF was developed using Cleanfile software. The essential data necessary for monitoring the primary and secondary endpoints are identified and managed at regular intervals throughout the trial. Data are monitored by the data management team of the data-management department of University Hospital of Lille by using the predefined rules, and queries will be automatically edited. Finally, an overall automated monitoring is done by the data manager at the end of the data entry. In case of discrepancies, queries will be edited to resolve the problems encountered.

### Sample Size Estimates

Based on the literature (19) and the data from the Endovascular Treatment in Ischemic Stroke (ETIS) registry, we expect an ICH rate of 40% in reperfused subjects with a conventional BP control during 24 h following reperfusion (SBP between 130 and 185 mmHg). We assume that a tight BP control during 24 h following reperfusion (SBP between 100 and 129 mmHg) will be associated with an absolute decrease of 15% (corresponding to a hemorrhage rate of 25%). To detect this effect size, with a two-sided test at the 0.05 level of significance and a power of 80%, 152 subjects per arm will be required. To account for an anticipated rate of 5% of uninterpretable follow-up CT scan, we planned to include a total of 320 subjects (160 per arm).

### Statistical Analyses

Statistical analyses will be independently performed by the Biostatistics Department of Lille University. Data will be analyzed using SAS software (SAS Institute Inc., Cary, NC, USA), and all statistical tests will be performed with a 2-tailed alpha risk of 0.05. Baseline characteristics will be described for each treatment group; categorical variables will be expressed as frequencies and percentages and quantitative variables will be expressed as means ± standard deviation or medians (interquartile range) for non-Gaussian distribution. Normality of distributions will be assessed graphically and by using the Shapiro–Wilk test. No formal statistical comparisons of baseline characteristics will be done; clinical importance of any imbalance will be noted. All analyses will be performed using all randomized participants based on their original group of randomization, according to the intention-to-treat principle. A per-protocol analysis will be considered only for the primary endpoint as a secondary analysis. Per-protocol population will include all randomized patients excluding those with major protocol violations (i.e., patients without reperfusion after EVT, patients with SBP <130 mm Hg at inclusion, patients not achieving their assigned SBP target). The final report will be written, based on the CONSORT statement recommendations.

#### Primary Outcome

The ICH rate on CT at 24–36 h will be reported in each arm, and the primary efficacy analysis will be conducted using a mixed logistic regression model including the center as a random effect and the randomization stratification factor (intravenous alteplase) as a fixed effect. The adjusted odds ratio (OR) will be derived from this model as the treatment effect size (experimental relative to control strategy). We will also calculate adjusted absolute and relative risk differences as effect sizes from the marginal probabilities of ICH rate, estimated by the previous mixed logistic regression model using the method described (20). Since we did not expect missing data on the primary endpoint, no imputation procedure will be applied for primary efficacy analysis. In case of missing data (whatever the reason), a sensitivity analysis will be conducted after handling missing primary endpoint values by a multiple imputation procedure. The imputation procedure will be performed using the main baseline characteristics and treatment group. Missing values will be imputed under a missing at random assumption by using the regression switching approach (chained equation with *m* = 10 imputations) with the predictive mean matching method for quantitative variables and logistic regression models (binary, ordinal, or polynomial) for categorical variables (21). Treatment effect estimates obtained in multiple imputed datasets are combined using Rubin's rules (22).

As exploratory analyses, heterogeneity in treatment effect size on primary outcome across key subgroups will be evaluated by including the corresponding multiplicative interaction terms in the multivariate mixed logistic regression models. From these models, treatment effect sizes (adjusted OR) will be estimated in each subgroup. The following key subgroups are investigated:

- Prior use of intravenous alteplase (yes vs. no)
- Age (≤ 80 years vs. >80 years)
- Baseline site of occlusion on vascular imaging [isolated middle cerebral artery (MCA) vs. tandem MCA/internal carotid artery (ICA)].

#### Secondary Outcomes

Secondary binary outcomes (symptomatic ICH, parenchyma hematoma type 2, favorable outcome, 90-day all-cause mortality) will be also analyzed similarly to the primary efficacy analysis.

The secondary ordinal outcome [distribution of 90-day after combining scores of 5 and 6 (23)] will be described by the median (interquartile range) for each treatment group and compared using a mixed ordinal logistic regression model including the same fixed and random effects that is used for primary efficacy model. The adjusted common OR per 1-point improvement will be calculated as the treatment effect size.

The change in NIHSS score at 24 h and infarct volume will be calculated and compared between the two treatment groups using the constrained longitudinal data analysis (cLDA) model proposed by Liang and Zeger (19, 20, 24) including the same fixed and random effects as in the primary efficacy model. This model will be used in view of the potential advantages of the cLDA compared to the conventional longitudinal analysis of covariance (ANCOVA) model. In the cLDA, both the baseline and post-baseline values will be modeled as dependent variables using a linear mixed model (using an unstructured covariance pattern model), and the true baseline means will be constrained to be the same for the two treatment groups. The between-group mean differences in 24-h change in NIHSS and infarct volume will be estimated by the time-by-arm interaction as treatment effect size. If the assumption of normality of model residuals is not satisfied (even after log-transformation), nonparametric analysis will be used; absolute changes between baseline and 24 h will be calculated and compared between the two treatment groups using nonparametric analysis of covariance adjusted for baseline values (25).

The other secondary quantitative outcome (stroke volume at follow-up CT scan) will be analyzed using a mixed linear regression model including the same fixed and random effects as the primary efficacy model; the between-group mean differences in stroke volume will be derived from the model as effect size. In case normality of model residuals is not satisfied (even after a logarithmic transformation), nonparametric analysis will be used; stroke volume will be compared using the Mann–Whitney *U* test.

Adverse-related serious adverse events (overall and individual events rates based on subject counts and not on event counts) will be evaluated descriptively in each treatment group, as well as the rate of patients with mean BP levels during BP management <80 mmHg without formal statistical comparison.

## Discussion

The BP TARGET Trial will be the first randomized controlled trial, with PROBE design, designed to compare the effect of two SBP target strategies, after EVT reperfusion, on ICH. We hypothesized that an SBP target of 100–129 mm Hg will be associated with a decreased risk of ICH. ICH on CT at 24 h was chosen as the primary outcome, considering the evidence supporting that elevated SBP is associated with ICH (10), a consequence of reperfusion injury, and associated with a worse prognosis. Additionally, recent evidence has shown that patients with mean SBP between 101 and 120 mmHg have lower odds of symptomatic ICH, when compared to those with SBP > 140 mmHg (13). Obviously, achieving BP control strategies in subjects with persistent arterial occlusion may not have the same consequence as that in subjects with complete reperfusion. In this context, we choose to study a homogeneous population with documented reperfusion of the anterior circulation.

Retrospective data from AIS subjects treated with EVT reveal a different impact of SBP values based on the reperfusion status (10). In fact, in subjects with successful reperfusion, maximum SBP correlates with worse functional outcome and ICH occurrence (10). Still, the observed relationship between BP and prognosis may be due to the fact that patients with a larger volume of brain ischemia have greater elevations in BP (26). Thus, BP elevation may be a marker of poor outcome, rather than the cause. Although analyses will include stratification on age, tandem lesions, or in participants receiving IVT, these are only exploratory and not designed to have the power to address the effect of the 100–130 mm Hg SBP target. In addition, we cannot exclude a differential effect due to the drug (e.g., reduced BP variability with calcium channel blockers compared to beta blockers).

## Summary and Conclusions

No previous head-to-head randomized trials have directly compared the efficacy of different SBP targets after EVT reperfusion. This prospective trial aims to determine whether a target SBP < 130 mm Hg after EVT reperfusion can reduce the risk of ICH.

## Ethics Statement

The studies involving human participants were reviewed and approved by Comité de Protection des Personnes Sud Méditerranée IV. The patients/participants provided their written informed consent to participate in this study.

## Author Contributions

MM and JS drafted the manuscript and designed the study. SR, BG, BL, IS, BM, and MO acquired the data. EH, PR, MP, RB, AY, LS, J-MO, JBe, and JBr made critical revisions of the manuscript. JL and AD wrote the statistical analysis plan and cleaned and analyzed the data.

## Conflict of Interest

RB and MP declare institutional fees for teaching presentations from Stryker, MicroVention, Balt. MM declares institutional fees for teaching presentations from Boerhinger Ingelheim, Medtronic, Amgen, and consulting fees from Boerhinger Ingelheim, Acticor Biotech. The remaining authors declare that the research was conducted in the absence of any commercial or financial relationships that could be construed as a potential conflict of interest.
